# Variants of 11β-hydroxysteroid dehydrogenase (*HSD11B*) gene type 1 and 2 in Chinese obese adolescents

**DOI:** 10.1186/1687-9856-2013-S1-P92

**Published:** 2013-10-03

**Authors:** Zou Chao Chun

**Affiliations:** 1 The Children's Hospital Of Zhejiang University School Of Medicine, China

## Objective

To investigate the relationship between 11β-hydroxysteroid dehydrogenase (*HSD11B*) gene type 1 and 2 and obesity in Chinese child.

## Methods

A total of 400 obese and 200 healthy adolescents were enrolled as obese and control groups.Seven tagged SNPs in *HSD11B1* (rs4393158, rs2235543, rs10082248, rs10863782, rs2236903, rs2298930, rs4545339) and 4 variants in *HSD11B2* gene (rs28934592, rs28934591, rs28934594 and rs28934593) were measured by automated platform MassArray.

## Results

The rs28934592 in *HSD11B2* and rs10863782 in *HSD11B1* were excluded as false positive or HWE P<0.05. Moreover, one allele type was found in the other 3 locations of *HSD11B2*. The minor allele frequency of rs2235543 and rs10082248 were higher in patients than these in controls (P=0.045, P=0.041, respectively). The rs10082248, rs2298930 and rs4545339 were associated with the risk of obesity in the recessive model (P<0.05, respectively). Moreover, the total cholesterol in patients with GG or AG genotype was significantly higher than that in patients with AA genotype in rs10082248. The rs4393158 was associated with the hypertension inlog-additive model test (P=0.037), and glucose abnormal and hypercholesteremiaindominant model test (P<0.05, respectively), while the rs2235543 was associated with hypercholesteremiainoverdominant model test (P=0.017).

## Conclusion

The polymorphism of *HSD11B1*may be a cause of childhood obesity, or even associated with the complication of childhood obesity. However, variants of HSD11B2 may be not a cause of obesity.

